# Evolution of Rice Storage Quality and Underlying Microstructural Mechanisms Under Varying Nitrogen Fertilization Application Levels

**DOI:** 10.3390/foods15101793

**Published:** 2026-05-19

**Authors:** Fei Wen, Jiahui Qi, Haimiao Yang, Wenbin Gu, Chenyu Rong, Jing Chen, Feifei Li, Xiangqian Zhao

**Affiliations:** College of Advanced Agricultural Sciences, Zhejiang Agriculture and Forestry University, Hangzhou 311300, China; 13398342644@163.com (F.W.); 19850507729@163.com (J.Q.); 15395718974@163.com (H.Y.); 15167138202@163.com (W.G.); rongchenyu@zafu.edu.cn (C.R.); jc@zafu.edu.cn (J.C.); lifei-fei@163.com (F.L.)

**Keywords:** *indica*-*japonica* hybrid rice, nitrogen fertilization, storage duration, quality stability

## Abstract

Nitrogen fertilizer application rate and storage duration are critical agronomic and environmental factors affecting rice quality stability. The milling appearance, eating and nutritional quality, physicochemical properties, microstructure, and volatile metabolic profiles during long-term storage were investigated using three *indica*-*japonica* hybrid cultivars at four nitrogen fertilizer application levels. High nitrogen fertilizer application (300 kg hm^−2^) promoted an over-filled protein matrix and induced structural defects such as micropores in starch granules, which acted as “trigger points” for accelerated aging. Specifically, storage duration was the dominant factor reshaping volatile profiles and lipid degradation, but high nitrogen amplified these effects by promoting lipid oxidation and the accumulation of off-flavor compounds. Correlation analysis revealed that gel consistency (GC) is a core determinant of eating quality, exhibiting significant negative correlations with amylose content, setback, hardness and fatty acid values, while showing positive correlations with peak viscosity, breakdown value, and adhesiveness. All correlation patterns collectively contributed to the deterioration of rice eating quality after storage, indicating GC might be served as an indirect indicator for evaluating rice deterioration and applied in the breeding of rice varieties with improved storage tolerance. Microstructural analysis via SEM high nitrogen induced distinct cultivar-specific deterioration characteristics after 12 months storage.

## 1. Introduction

Rice is a major staple food crop globally, and its stable supply is crucial for maintaining global food security [1]. With the improvement of living standards, cooking and eating quality (CEQ) has become an important indicator of rice commodity value, which was affected by intrinsic ingredients in rice, such as starch, protein, lipid and other components [2,3,4]. In general, cooked rice with high amylose content (AC) exhibits slower starch digestibility, but it tends to possess a dry and flaky texture, subsequently resulting in poorer CEQ compared with low-AC rice [5,6]. Some studies have demonstrated that rice with low protein content (PC) typically exhibits superior CEQ, whereas rice with high PC tends to have increased grain hardness, particularly when the protein content exceeds 7% [7,8,9]. Protein fractions, including prolamin, albumin, glutelin, and globulin also affect CEQ by influencing starch hydration rates during cooking [10,11]. Usually, rice varieties with lower glutelin and prolamin contents but higher albumin content present better eating quality, which is attributed to the significant negative correlation between albumin content and AC [12,13]. Lipids, binding with amylose and amylopectin to form complexes, interact with each other, thereby affecting gel consistency (GC) and the viscosity of rice elasticity and texture during starch gelatinization [14]. Rice with higher lipid content had a brighter luster and better eating quality [15].

As one of the most critical nutrients for rice growth and development, nitrogen not only plays a key role in grain yield but also acts as a vital agronomic factor that directly regulates rice quality [16]. Appropriate nitrogen supply promotes grain filling and dry matter accumulation, thereby enhancing both milling and appearance quality of rice. However, excessive nitrogen fertilizer application often results in uneven grain filling, leading to increased percentage of grain with chalkiness (PGWC) and chalkiness degree (CD) [17,18,19,20], and may also exert adverse effects on rice CEQ [21,22], since it decreased the average and maximum grain filling rate and lengthened the active period of grain filling [23]. Many studies demonstrated that the PC was enhanced with nitrogen fertilizer application levels, whereas AC was reduced accordingly [24,25,26]. In particular, nitrogen fertilizer application at the panicle differentiation stage can increase rice grain hardness, decrease taste score, and deteriorate pasting properties. These changes were mainly caused by elevated PC alongside reduced starch and crude fat contents, and impaired starch granule development; in addition, high nitrogen application promoted protein accumulation around starch granules during cooking, thereby restricting starch gelatinization and deteriorating eating quality. which ultimately lead to the degradation of CEQ [27,28,29,30]. These findings indicate that nitrogen fertilizer affects rice quality not only by changing protein and starch contents but also by regulating starch–protein interactions and starch structural characteristics. Therefore, adopting targeted cultivation strategies tailored to specific rice varieties could effectively satisfy the dual demands of high yield and superior quality in rice production [31].

Rice production is limited by seasonal and regional factors, making it difficult to meet the continuous and widespread consumption demand for rice. In order to strengthen food security, implement strategic reserve policies, improve disaster response capabilities, and ensure basic supply, it is crucial to maintain stable rice production and quality [28]. Global rice ending stocks, a core indicator reflecting annual rice storage capacity, are projected to reach a record high of 219.3 million tons (milled basis) in the 2025/26 marketing year according to the Food and Agriculture Organization (FAO), highlighting the critical importance of maintaining rice storage quality for global food security [29]. Notably, the decline in rice quality due to aging during storage not only directly affects the marketability of rice but can also pose risks to human health [30]. As a living organism, rice undergoes various degrees of metabolism of metabolic activity via respiration throughout the storage period, triggering irreversible alterations in its intrinsic quality [32]. Specifically, starch is gradually decomposed into dextrins and maltose under the action of amylase, accompanied by an increase in reducing sugars; proteins are broken down into peptides and amino acids through the synergistic action of proteases and peptidases; and lipids are hydrolyzed into glycerol and free fatty acids [33]. These biochemical changes collectively constitute one of the key mechanisms leading to the deterioration of rice quality. In addition, these changes also weaken the structural integrity of rice grains, reduce the head rice rate and increase the broken rice rate. Meanwhile, they alter the optical properties, resulting in elevated PGWC [34]. With the extension of storage time, the CEQ of rice generally deteriorates, accompanied by elevated hardness, decreased adhesiveness (AD), and weakened flavor characteristics [35,36].

During aging, total starch, fat, and protein contents decreased, whereas amylose content increased; meanwhile, the short-range ordered structure of starch and the secondary structure of proteins gradually deteriorated, resulting in reduced taste value and lower 2-acetyl-1-pyrroline content [37]. 12 months of storage decreased starch crystallinity, granule size, amylose content, and protein content, while increasing the proportion of amylopectin A chains in ratoon rice [38]. Long-term storage caused degradation of rice starch molecules, reduced double helices, short-range ordered structures, and crystalline structures, and induced apparent erosion of starch granules [39]. These results suggest that starch structural degradation is a major contributor to the deterioration of rice quality during storage. This aging process is mediated by the combined effects of starch structure rearrangement, enhanced protein intermolecular interactions, and lipid oxidation [40,41].

Beyond physicochemical quality, flavor characteristics are critical determinants of consumer acceptance. Rice aroma is formed by the combined effects of various volatile compounds, such as aldehydes, alcohols, and ketones. During storage, lipid oxidation, particularly the formation of pentanal and hexanal, is the primary cause of off-flavor development in aged rice [42]. Volatile organic compounds changed markedly during rice storage, especially aging-related compounds such as hexanal and 2-pentylfuran, and that lipid oxidation and antioxidant activity were closely associated with varietal differences in storage tolerance [43]. Prolonged storage reduced antioxidant capacity and altered the ordered structure of starch in traditional aromatic white and black rice, indicating that oxidative deterioration and starch structural changes jointly contribute to quality decline during storage [44]. Accordingly, storage duration has emerged as a crucial factor determining the stability of rice quality during storage.

In addition, with continuous breakthroughs in breeding technology, *indica*-*japonica* hybrid rice has been extensively deployed in rice production, owing to its outstanding combination of the high yield potential of *indica* rice and the superior eating quality of *japonica* rice [45]. *Indica*-*japonica* hybrid rice is characterized by large biomass, big panicle size and high yield potential (over 12 t hm^−2^), and thus requires more nitrogen to support dry matter accumulation and the formation of large panicles. Therefore, these varieties present remarkable complexity in grain quality formation. In these practical production systems, the initial grain quality foundation formed by field nitrogen management does not remain unchanged, but continuously evolves throughout the storage process, suggesting that significant interactive effects may exist between pre-harvest nitrogen fertilization and postharvest storage. CEQ of *indica*-*japonica* hybrid rice deteriorated more rapidly than that of *indica* and *japonica* rice during storage, particularly in terms of fatty acid value, cooked rice appearance, cooked rice texture and comprehensive taste value [46].

Recent studies have further confirmed that the response of rice quality to nitrogen application is highly dependent on cultivar type and grain composition. For example, nitrogen levels significantly affected the comprehensive quality of rice in South China, and that the optimal nitrogen level differed between hybrid *indica* rice and inbred *japonica* rice [47]. Although a large number of previous studies have investigated the individual influences of nitrogen application and storage time on rice quality, the interactive effect between these two factors unreported. To date, comprehensive studies focusing on the coupled effects of nitrogen application rates and storage duration on the quality traits of *indica*-*japonica* hybrid rice are still insufficient. Moreover, the quality response patterns of different *indica*-*japonica* hybrid rice varieties to the interaction of nitrogen levels and storage time remain largely unclear, which merits further systematical exploration.

Therefore, the dynamic changes in milling, appearance, nutritional quality and CEQ during storage on *indica*-*japonica* hybrid rice under set gradient nitrogen fertilizer application levels were systematically investigated in this study. Meanwhile, the starch microstructure, volatile compounds and amylopectin chain length (ACL) distribution of rice grains were determined. Correlation analysis was further performed to clarify the relationships among key quality indicators. This study aims to elucidate effects of nitrogen management and postharvest storage on the quality deterioration and variation in *indica*-*japonica* hybrid rice, thereby providing a theoretical reference for optimizing nitrogen fertilization regimes and formulating scientific postharvest storage technologies.

## 2. Materials and Methods

### 2.1. Materials and Field Management

Three main cultivated cultivars of *indica*-*japonica* hybrid rice, Jiafengyou 2 (JFY2), Yongyou15 (YY15), and Zhejingyou 1578 (ZJY1578) in Zhejiang Province were selected in this study. The field experiment was conducted in 2023 at Senle Agricultural Development Co., Ltd. in Yuhang District, Hangzhou City (30.295 °N, 119.863 °E), China. The experimental site has a typical subtropical monsoon climate. The experimental soil was the plow layer soil, and its basic physicochemical properties are as follows: organic matter content 24.53 g kg^−1^, total nitrogen 2.08 g kg^−1^, available phosphorus 38.4 mg kg^−1^, and available potassium 95.3 mg kg^−1^.

The field experiment was designed using a split-plot design with two factors. The main factor was nitrogen fertilizer application level, with four levels: N5, N10, N15 and N20 representing pure nitrogen application rate at 75, 150, 225 and 300 kg hm^−2^ respectively. The subplot factor was rice variety, including JFY2, YY15, and ZJY1578. The rice varieties were randomly arranged in the subplots to eliminate the systematic error caused by non-random distribution of varieties with three replications. All treatments were arranged in a randomized complete block design. The plot size and planting density were strictly standardized, with 18 hills in row and 14 hills in column, and a spacing of 8 inches between rows and 11 inches between columns, respectively. Nitrogen fertilizer in the form of urea (N ≥ 46%) was applied in stages according to a ratio of 5:3:2 for basal fertilizer, tillering fertilizer, panicle fertilizer respectively. Phosphorus (80 kg hm^−2^ as P_2_O_5_) and potassium (50 kg hm^−2^ as KCl) were applied with nitrogen before transplanting as basal fertilizer. Other field management was performed according to local farming practices.

Paddy on each plot was harvested separately at maturity on morning after the dew evaporation, and sun-dried to a moisture content of approximately 13.0–13.5%. During sun drying, the paddy was stirred and measured moisture content every hour to minimizing intense respiratory and physiological activities. Dried samples were equilibrated in nylon mesh bags at room temperature for 1 month. A total of 2 kg rice paddy from each plot was placed in mesh bags and stored in a controlled climate chamber for simulated storage. The temperature was set at 25 ± 1 °C, with relative humidity (RH) maintained at 70 ± 5%. Samples were taken at 6 and 12 months (6M and 12M) after storage for quality analysis, respectively. Fresh paddy was treated as check.

### 2.2. Grain Quality Analysis

A total of 125 g rough paddy was dehulled and polished with a milling time of 35 s using LTJM-160 Fengsu Rice Polisher (Oasis Testing Instrument Co., Ltd., Shanghai, China). Percentage of grain with chalkiness (PGWC) of polished grains was measured manually. Milled rice yield (MRY) was determined from the weight of polished grains recovered in 125 g rough rice. Head rice, grains length greater than or equal to 3/4 total length, was separated from milled rice manually. The weight of head rice was used to calculate head rice yield (HRY) relative to the original 125 g rice paddy. Amylose content (AC) was measured by the standard iodine colorimetry method described in ISO 6647-2-2011 [48]. Gel consistency (GC) was determined following the optimized method described by Zhang et al. [49]. Alkali spreading value (ASV) was measured using 1.70% (*w*/*v*) potassium hydroxide (KOH, Sinopharm Chemical Reagent Co., Ltd. Shanghai, China) solution at 30 °C for 23 h [50]. All indicators were measured in triplicate.

Crude protein and crude fat content were measured using a near-infrared analyzer (FOSS Analytical A/S, Hilleroed, Denmark), with calibration performed using known standard samples before measurement. Each sample was measured in triplicate. Fatty acid value was determined according to the national standard GB/T 15684-2015 [51] “Grain and Oil Inspection-Determination of Fatty Acid Value in Grains and Products Using an Automatic Titration Analyzer”.

Rheological properties of starch structure were determined using a Rapid Visco Analyzer (RVA-Tec Master, Perten Instruments AB, Stockholm, Sweden) based on the manufacturer’s instructions. Exactly 3.00 g rice flour (sieved through a 100-mesh screen) was weighed and placed in a special aluminum can, to which 25.0 mL distilled water was added. The test procedure was as follows: maintain 50 °C for 1 min, then heat at 12 °C/min to 95 °C and hold for 2.5 min, followed by cooling at 12 °C/min to 50 °C and holding for 2 min. The peak viscosity, hot paste viscosity, cold paste viscosity, breakdown, setback, and gelatinization temperature were recorded [52]. Texture characteristics were measured using a texture analyzer (TMS-PRO, Food Technology Corporation, Sterling, VA, USA), with the following parameters: deformation 60%, test speed 60 mm/min, trigger force 0.2 N, compression interval time 2 s, and probe return height 7 mm [53]. Each sample was measured in triplicate.

### 2.3. Starch Microstructure

A total of 10 g of rice flour was placed into a 50 mL centrifuge tube, and alkaline protease was added at a ratio of 50 mg/g. Subsequently, 25 mL of NaOH solution (pH 10) was added, and the mixture was incubated at 42 °C on a shaker for 24 h. The starch slurry was then passed through a 200-mesh sieve, and the filtrate was centrifuged at 4000× *g* for 20 min. The supernatant was discarded, and the yellow layer on the starch surface was carefully removed. The starch precipitate was resuspended in deionized water and centrifuged again at 4000× *g* for 20 min. This washing step was repeated 3 times to remove residual impurities. The purified starch was sequentially washed 3 times with 95% ethanol, chloroform–methanol mixture (*v*/*v* = 1:1), and methanol–acetone mixture (*v*/*v* = 1:1, Sinopharm Chemical Reagent Co., Ltd., Shanghai, China) to remove lipids. The extract was dried at 70 °C (stirred with a glass rod when semi-dry), passed through a 200-mesh sieve, and collected as purified starch [54].

Starch chain-length distribution was determined using high-performance anion-exchange chromatography with pulsed amperometric detection (HPAEC–PAD). Briefly, 10 mg of purified starch was gelatinized in a boiling water bath and subsequently debranched with isoamylase (1400 U, Jingke Chemical Technology Co., Ltd, Shanghai, China) for 24 h. The analysis was performed using a Thermo ICS-5000 ion chromatography system (Thermo Fisher Scientific, Waltham, MA, USA) equipped with a Dionex™ CarboPac™ PA200 column. The flow rate was set at 0.4 mL min^−1^, and a gradient elution of 0.2 M NaOH and 0.2 M sodium acetate was applied to separate glucan chains with different degrees of polymerization (DP) [55].

The microstructure of starch granules was observed using a scanning electron microscope (SEM, Hitachi High-Tech Corporation, Tokyo, Japan). Samples were dried at 40 °C for 12 h, mounted on sample stubs, and sputter-coated with gold. Observations were conducted at a magnification of 3000×, and representative images were recorded to characterize the surface morphology of starch granules.

### 2.4. Rice Volatile Compounds

Volatile compounds were analyzed using headspace solid-phase microextraction coupled with gas chromatography–mass spectrometry (HS-SPME–GC–MS, Shimadzu Corporation, Kyoto, Japan). Approximately 2 g of brown rice flour was weighed into a 20 mL headspace vial, and 20 μL of 2-octanol solution (internal standard) was added. Extraction was performed at 80 °C for 40 min, followed by desorption for 5 min. GC–MS conditions: The injector temperature was set at 250 °C in splitless mode. Helium was used as the carrier gas at a constant flow rate of 1.0 mL min^−1^. The oven temperature program was as follows: initial temperature of 45 °C (held for 1 min), increased to 150 °C at 4 °C min^−1^ (held for 4 min), and then raised to 210 °C at 3 °C min^−1^ (held for 2 min). The MS interface temperature was 280 °C. The ion source was an electron ionization (EI) source operated at 70 eV, with an iron source temperature of 230 °C. Full-scan acquisition was performed over a mass range of 50–550 amu, with 2 min solvent delay. Signals were processed using unknown analysis software with automatic integration. Compounds were identified by spectral deconvolution and comparison with the NIST20.L library (matching degree > 70%). Relative contents of each component were calculated using the area normalization method [56].

### 2.5. Data Processing and Analysis

Basic data processing was performed using Microsoft Excel 2019 (Microsoft Corporation, Redmond, WA, USA). According to the division of variation sources in the split-split-plot design, a three-factor ANOVA model was constructed for the sum of squares (SS), mean square (MS), F and *p* value of each variation source calculation, and then Duncan’s new multiple range test (DMRT) was used for multiple comparisons of differences among treatments, with the significance level set at *p* < 0.05. Statistical analysis was conducted using IBM SPSS 22.0 (IBM Corporation, Armonk, NY, USA). Significant differences were evaluated using Duncan’s multiple range test (*p* < 0.05). Graphs were generated using Origin 2024 (OriginLab Corporation, Northampton, MA, USA) and SIMCA 14.1 (Sartorius AG, Göttingen, Germany).

## 3. Results

### 3.1. Processing and Appearance Quality

Three-way analysis of variance revealed that storage duration, variety, and nitrogen application rate all significantly affect the MRY, HRY, and PGWC (Table S1). Predominant effects on MRY and HRY were controlled by varieties and nitrogen application rate, while PGWC was majorly affected by variety and storage duration. Notably, most interactions among these three factors also reached significant or highly significant levels for afore mentioned quality indicators, which indicates that the regulatory effect of nitrogen fertilizer application on grain quality is co-modulated by both genotypic characteristics of the variety and postharvest storage duration.

Obvious genotypic differences were detected among the three tested varieties. Milled rice yield (MRY) of three varieties increased significantly with increasing nitrogen fertilizer application levels, and reached the maximum under N20 treatment (Table 1). With prolonged storage, MRY showed a decreasing trend, and was significantly lower at 12 months of storage (12M) than newly harvested samples (0M). Among the three varieties, YY15 exhibited the highest and most stable MRY, followed by JFY2 and ZJY1578.

The head rice yield (HRY) varied with nitrogen fertilizer application levels. JFY2 exhibited the highest HRY under N5, whereas YY15 and ZJY1578 reached their maximum HRY under N20. Compared with freshly harvested paddy, YY15 showed increased HRY across three lower nitrogen treatments at 6 months of storage (6M), followed by a decline at 12 months (12M). JFY2 under N10, N15, and N20, as well as ZJY1578 under N20, displayed a trend similar to that of YY15. For the other treatments, HRY exhibited a continuous decreasing trend with prolonged storage and was significantly lower at 12M.

The regulatory effect of nitrogen fertilizer on PGWC varied among rice varieties. The PGWC of JFY2 was significantly higher at N20 than at the other three nitrogen rates. For YY15, the PGWC at N5 was significantly lower than that at the other three nitrogen levels. The PGWC of ZJY1578 was similar between N5 and N20, and higher than that at the other two treatments. Compared with fresh samples, the PGWC of JFY2 remained relatively stable at 6M, but increased rapidly at 12M, at least 30% higher than that of 6M across 4 nitrogen treatments. For YY15, PGWC decreased significantly at 6M and then increased sharply, except under the N5 treatment. In general, the PGWC of ZJY1578 rose continuously with extended storage.

Overall, elevated nitrogen input improved milling quality represented by MRY but impaired appearance quality by increasing chalkiness. Prolonged storage led to consistent quality deterioration, including reduced milling quality and increased chalkiness.

### 3.2. Nutritional Quality

**Figure 1:**
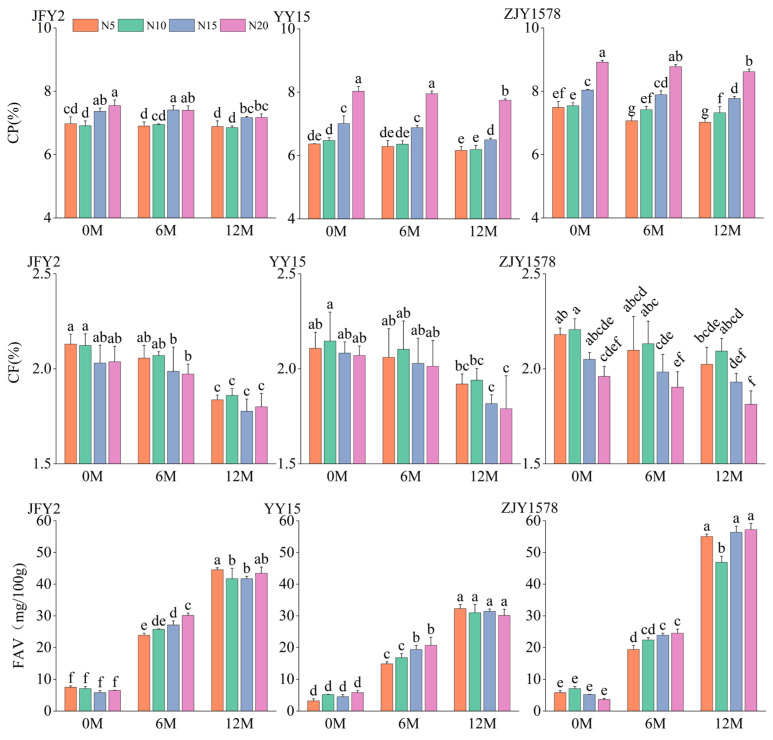
Effects of nitrogen fertilizer application rate on nutritional quality of *indica*-*japonica* hybrid rice under different storage durations. Note: JFY2, YY15, and ZJY1578 represent Jiafengyou 2, Yongyou 15, and Zhejingyou 1578, respectively. In the figure, N5, N10, N15, and N20 represent 75, 150, 225, and 300 kg hm^−2^ of pure nitrogen, respectively. 0M, 6M, and 12M represent storage durations of 0, 6, and 12 months, respectively. Different lowercase letters on the bars indicate significant differences at the 0.05 level within each cultivar (Duncan’s range test, *n* = 3). CP, CF, FAV represent crude protein content, crude fat content, fatty acid value, respectively. The same applies hereafter.

The analysis of variance revealed that nitrogen fertilizer application level and cultivar exerted major effects on crude protein content of rice grain (Table S2). The crude protein content of the three varieties significantly increased with the increase nitrogen fertilizer application level across all storage stages (Figure 1). The highest content was observed under the N20 treatment, demonstrating a clear positive regulation by nitrogen fertilization. Although slight fluctuations in crude protein content were induced by the significant storage duration during the 12-month storage period, the overall variation trend in response to nitrogen fertilizer levels remained consistent. This indicates that nitrogen fertilizer application rate acts as the core factor determining the protein accumulation in *indica*-*japonica* hybrid rice, and this regulatory effect remains stable throughout postharvest storage.

The mean square of storage duration was markedly higher than that of other factors, indicating that postharvest storage served as the dominant factor regulating crude fat variation in rice grains (Table S2). Nitrogen application rate exhibited a negative regulatory effect on crude fat content, that is, increasing the nitrogen application rate reduced the initial crude fat content in rice grain (Figure 1). Plant lipid synthesis uses acetyl-CoA, a key intermediate of carbohydrate metabolism, as the core substrate [57]. Accordingly, the synthesis and accumulation of grain lipids depend largely on carbohydrate reserves and are tightly regulated by carbon metabolic pathways. Crude fat content of the three varieties significantly decreased across all nitrogen application levels, which could be attributed to ongoing oxidation and hydrolytic degradation of lipid during storage. After 12 months of storage, the crude fat content under the high nitrogen treatment (N20) reached the lowest value. These results suggest that although high nitrogen fertilizer increased protein content, it reduced the relative proportion of lipids, and the storage process further accelerated the degradation and consumption of lipid substances.

The mean square of storage duration was significantly higher than that of other factors, indicating that postharvest storage was the leading factor regulating fatty acid value variation in rice grains (Table S2). The fatty acid value increased sharply with prolonged storage; after 12 months of storage (12M), all treatments were exhibited markedly higher values relative to the initial level (0M), with an increase of at least fourfold in all 4 nitrogen application rates (Figure 1). At the mid-storage period (6M), nitrogen application exerted a positive promoting effect on fatty acid value, whereby increasing nitrogen application significantly elevated the fatty acid value across three cultivars. This indicates that high nitrogen treatment accelerates lipid hydrolysis during the early to middle storage period. In terms of cultivar differences, YY15 maintained a relatively lower fatty acid values at 12M storage than the other two varieties, reflecting stronger storage tolerance. By contrast, ZJY1578 presented the most pronounced increase in fatty acid value, suggesting that its grain quality is more susceptible to storage induced deterioration.

### 3.3. Cooking and Eating Quality

**Figure 2:**
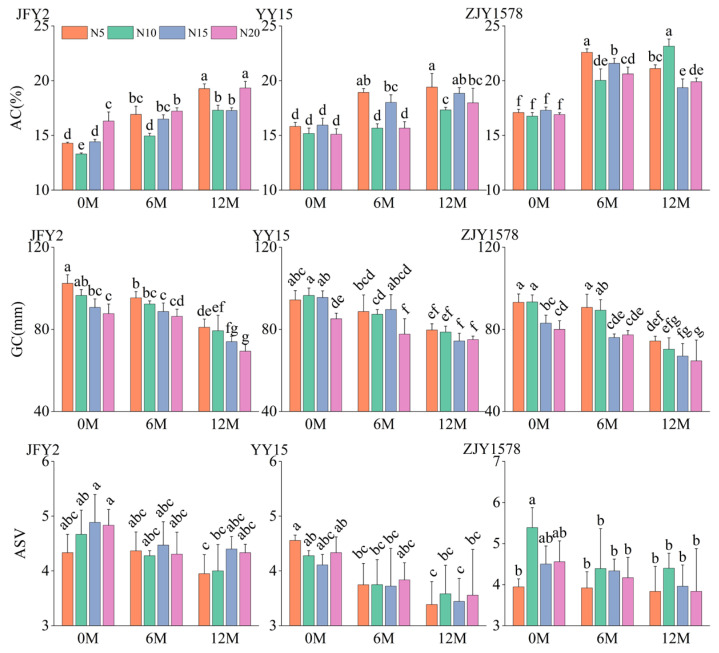
Effects of nitrogen fertilizer application level on cooking and eating quality of *indica*-*japonica* hybrid rice under different storage durations. Note: AC, GC and ASV represent amylose content, gel consistency and alkali spreading value, respectively. Different letters within one each cultivar represent significant differences at *p* < 0.05, *n* = 3.

Storage duration and cultivars were major two factors affecting amylose content (Table S3). Nitrogen fertilizer application level affected AC variation in JFY2, whereas no significant differences were observed in the other two cultivars. After 12 months of storage, AC significantly increased across all nitrogen treatments in 3 varieties, which suggests that long-term storage alters the physicochemical properties of rice starch and potentially increases the hardness of cooked rice (Figure 2).

Storage duration, variety, and nitrogen fertilizer application level all have highly significant effects on gel consistency (Table S3). With the extension of storage time, The GC of all treatments decreased significantly, indicating that storage duration gradually hardened the rice texture. Meanwhile, nitrogen fertilizer application level exerted a significant negative regulatory effect on GC. Within the same storage period, gel consistency declined markedly with the increase in nitrogen fertilizer application level, with the lowest GC value observed under the N20 treatment (Figure 2). These findings suggest that high-nitrogen fertilization not only increases grain protein content but also reduces the fluidity of the rice gel, thereby weakening the softness of eating quality. Furthermore, this adverse effect became more pronounced during long-term storage.

Storage duration and variety have highly significant effects on the rice alkali spreading value, while the effect of nitrogen application did not reach a significant level (Table S3). With the extension of storage time, ASV of all three varieties gradually decreased, indicating that long-term storage increased the starch gelatinization temperature and deteriorated the rice cooking properties (Figure 2). These finding indicated that gelatinization temperature, as an inherent physicochemical property of the variety, is less sensitive to cultivation practices such as nitrogen application and is primarily governed by genetic factors.

Except for peak viscosity (PV), other RVA parameters were primary controlled by postharvest storage. Nitrogen fertilizer application level and cultivars also exhibited highly significant effects on PV (Table S4). Among all nitrogen treatments, PV was the lowest under N20 across three cultivars, whereas YY15 exhibited the highest PV among the three cultivars (Table 2). In addition, within the same storage phase, as nitrogen fertilizer application increased, PV and breakdown values (BD) generally decreased, while the setback value increased. This suggests that high nitrogen fertilization altered the physicochemical structure of starch to a certain extent, rendering it more resistant to gelatinization and more susceptible to retrogradation during cooling, thereby contributing to a deterioration of eating quality. With the extension of storage time, the PV and BD of all treatments significantly decreased, while the setback and final viscosity (FV) significantly increased, indicating that storage duration significantly reduced the starch’s swelling capacity and thermal stability, and exacerbated the tendency toward retrogradation in rice.

The mean square of storage duration was significantly higher than that of other factors, indicating that postharvest storage served as the primary regulator of the hardness, springiness and gumminess variation. In addition, both storage duration and cultivars also exhibited significant effects on adhesiveness, cohesiveness, and chewiness (Tables S6 and S7). Hardness of fresh samples was not different across 4 nitrogen treatments, but which were increased dramatically with the extension of storage duration (Table 3). In general, compared with fresh samples, adhesiveness, cohesiveness and springiness were decreased quickly after 12M of storage. The gumminess of JFY2 was significantly increased across 4 nitrogen fertilizer application levels; however, that of ZJY1578 was decreased.

### 3.4. Relationships Among Tested Quality Traits

**Figure 3:**
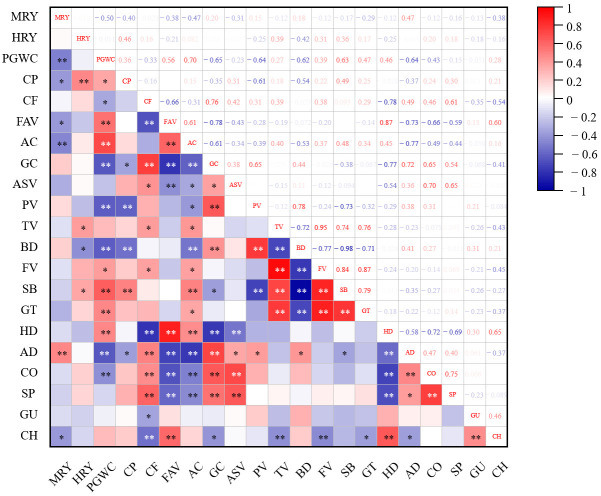
Correlation analysis of quality indexes of *indica*-*japonica* hybrid rice. Note: * and ** represent significant difference at *p* < 0.05 and *p* < 0.01 respectively.

The correlation heatmap elucidated the intrinsic relationships among rice grain quality, physicochemical properties, and TPA parameters (Figure 3). MRY showed significant positive correlations with AD, and negative correlation with PGWC, AC, CP, FAV and CH. HRY only exhibited a significant positive correlation with CP. PGWC exhibited strong positive correlations with AC, FAV, HD and SB, while presenting negative correlation with GC, AD, PV and BD. The correlation profile of AC with other traits was highly consistent with that of PGWC; by contrast, GC and CF showed relationship trends opposite to AC. CP had negative correlation with GC, AD and PV. Almost all starch pasting properties, TPA parameters, CF and FAV showed significant correlations. CF was positively correlated with GC, PV, BD and AD, and negatively correlated with FAV, SB, HD and CH, meanwhile FAV showed oppose relationship with above traits owing to its significant negative correlation with CF. BD showed a significant negative correlation with SB, reflecting the intrinsic trade-off between starch paste stability and retrogradation potential. Hardness, a core TPA index, exhibited strong correlations with starch properties, which was positively correlated with SB and negatively correlated with PV, BD, AC and GC. These results confirmed that starch retrogradation, regulated by AC and GC, was the dominant factors determining rice texture, especially hardness, during postharvest storage.

### 3.5. Microstructure and Mechanism of Starch

The cultivar was the major factor regulating short A chain (Fa) in amylopectin. Meanwhile, the interaction effects between storage duration and cultivar, cultivar and nitrogen fertilizer application level, as well as the three-way interaction among these factors also exerted significant effects (Table S8). In general, storage duration and cultivar were the predominant factors regulating B1 chains (Fb_1_), B2 chains (Fb_2_), B3 long chains (Fb_3_), and the average chain length of amylopectin (ACL), accompanied by significant interactions among three factors (Table 4). Nitrogen fertilization and storage exhibited inconsistent effects on the amylopectin chain-length distribution of different rice cultivars. At the N5 level, the Fa of JFY2 was the highest, while there were no significant differences among the other three nitrogen fertilizer treatments; in contrast, the performance of Fb_3_ was the opposite. After 12 months of storage, the Fa content of the N5 treatment decreased, whereas that of the other three nitrogen fertilizer treatments increased significantly, especially the N20 treatment. Compared with the fresh samples, after 12 months of storage, the Fa of YY15 increased significantly under N5, N10 and N15 treatments, while it decreased under N20 treatment; in contrast, the Fb3 decreased under all four nitrogen fertilizer treatments. With the increase of nitrogen fertilizer application level, the Fa content of ZJY1578 increased, while that of Fb3 decreased.

**Figure 4:**
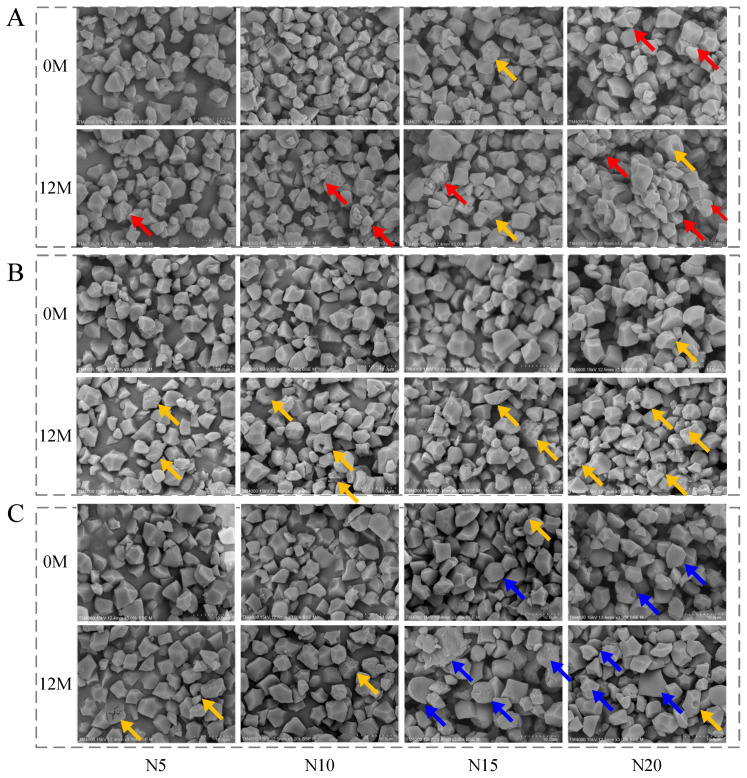
Morphology of starch granules of *indica*-*japonica* hybrid rice after storage under different nitrogen fertilizer application levels. Note: N5, N10, N15, and N20 in the figure represent pure nitrogen fertilizer application levels of 75, 150, 225, and 300 kg hm^−2^, respectively. 0M and 12M represent storage for 0 and 12 months, respectively. (**A**), (**B**), and (**C**) represent Jiafengyou 2, Yongyou 15, and Zhejingyou 1578, respectively. Red arrows: Indicate severe granule adhesion, blurred boundaries, and an over-filled protein matrix. Yellow arrows: Highlight honeycomb-like depressions and pitting pores on the granule surface. Blue arrows: Point to flattened granules with central collapse, structural cracks, and visible pores. Pictures were token by Hitachi TM4000 SEM at 15 kV accelerating voltage, 12.3 mm working distance, 3000× magnification and BSE (mixed mode) imaging.

The starch granule morphology of the tested cultivars under different nitrogen fertilizer application levels was observed via scanning electron microscopy (SEM) in both fresh (0M) and stored (12M) grains. The results indicated that nitrogen fertilizer not only enhanced the compactness of granule arrangement in fresh samples, but also induced distinct cultivar-specific deterioration characteristics during storage. As illustrated in Figure 4, with the increase in nitrogen fertilizer application level, all cultivars exhibited a tighter granule arrangement and reduced intergranular gaps. This phenomenon was mainly attributed to the excessive filling of the protein matrix promoted by high nitrogen fertilizer application level, which encapsulated and compressed the polyhedral starch granules. Notably, the N20 treatment already presented local depressions and micropore structures in fresh grains, indicating that high nitrogen might disrupt the coordination between starch and protein accumulation, leading to insufficient granule development and the formation of potential structural defects. After 12 months of storage, depressions, pores, depressions, and cracks appeared to varying degrees in all treatments, while the deterioration pathways induced by high nitrogen differed significantly among cultivars. Under high nitrogen conditions, JFY2 showed severe granule adhesion and blurred boundaries, which restricted the water absorption and swelling of starch and served as a key factor contributing to the decrease in breakdown viscosity. YY15 was mainly characterized by honeycomb-like depressions and pitting pores on the granule surface, suggesting higher sensitivity to chemical degradation during storage. In contrast, ZJY1578 flattened granules with central collapse, structural cracks, and visible pores, reflecting a loose internal structure that facilitates substance migration and accelerates structural deterioration during storage, thereby ultimately impairing the eating quality.

### 3.6. Volatile Compounds

**Figure 5:**
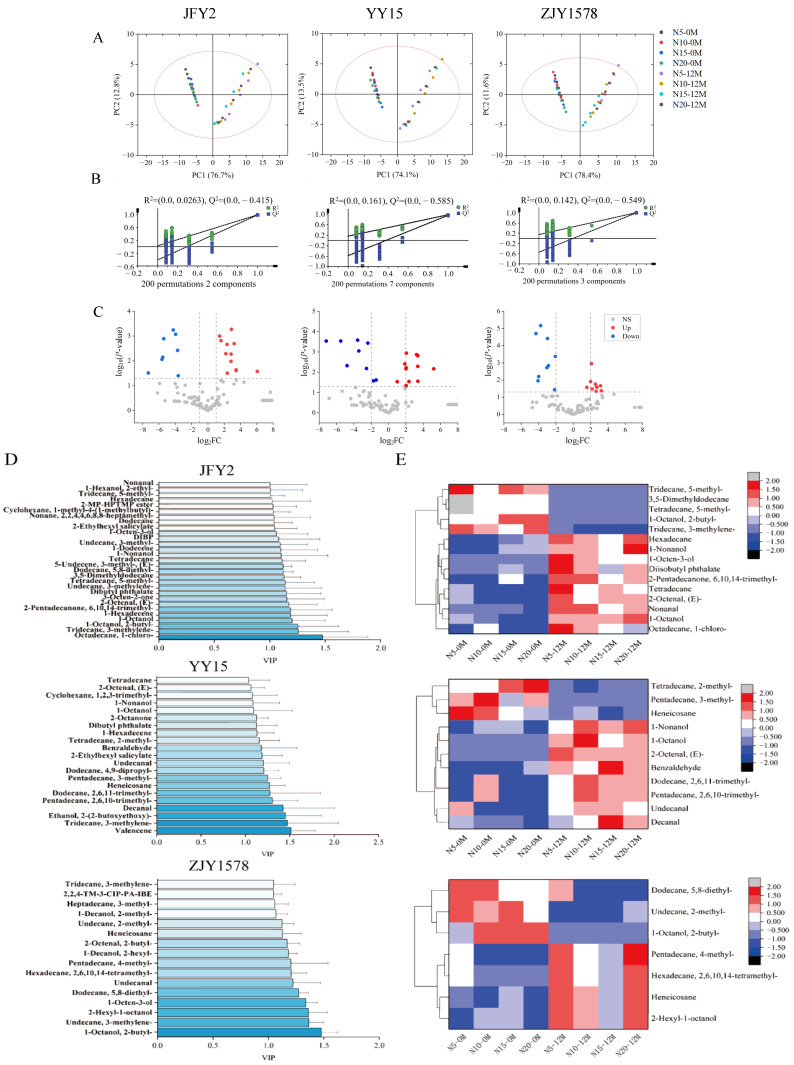
Profiles of volatile metabolites in *indica*-*japonica* hybrid rice after storage under different nitrogen fertilizer application levels. Note: (**A**) PCA score plot of volatile metabolites. (**B**) OPLS-DA permutation test plot. (**C**) Volcano plot of differential metabolites among different treatment groups. Red dots represent significantly up regulated metabolites, blue dots represent significantly down regulated metabolites, and gray dots indicate no significant difference (screening criteria: |log__2_FC| > 1 and *p* < 0.05). (**D**) Bar plot of substances with VIP > 1 in OPLS-DA model. (**E**) Hierarchical clustering heatmap of core differential volatile metabolites. The two vertical dashed lines indicate the fold-change thresholds of log_2_*FC* = ±1, and the horizontal dashed line indicates the significance threshold of *p* = 0.05, corresponding to −log_10_(*p*-value) = 1.30. Red dots represent significantly up-regulated components, blue dots represent significantly down-regulated components, and gray dots represent non-significant components.

The results of principal component analysis (PCA) indicated that samples with different storage durations were clearly separated along the PC1 axis, suggesting that storage time was the dominant factor driving the variations in volatile compounds (Figure 5A). In contrast, samples from different nitrogen treatments clustered closely within the same storage stage, demonstrating that nitrogen fertilizer application levels exerted a relatively weak influence on the overall profile of volatile components. Meanwhile, a certain separation trend was observed among cultivars, implying that genotype regulates volatile metabolite compounds.

To further enhance the discrimination between groups, an OPLS-DA model was applied (Figure 5B), which achieved satisfactory separation among all treatment groups. A 200-permutation test was conducted for model validation. The Q_2_ intercepts of all cultivars were below zero (JFY2: −0.415; YY15: −0.549; ZJY1578: −0.585), confirming no overfitting and verifying good stability and predictive capability of the model.

Volcano plot analysis revealed substantial changes in numerous volatile compounds before and after storage, predominantly showing upregulation (Figure 5C). This indicated an overall accumulation trend of volatile metabolites during storage, with differential compounds mainly concentrated in aldehydes and alcohols. According to the VIP screening results from the OPLS-DA model, key differential substances with VIP > 1 included nonanal, decanal, 1-octanol, 1-nonanol, and benzaldehyde (Figure 5D). These compounds contributed greatly to intergroup differences and represented the core variables distinguishing the samples.

Hierarchical clustering heatmap analysis further demonstrated that samples before storage (0M) and after storage (12M) were distinctly divided into two clusters at the overall metabolic level, reinforcing that storage duration was the primary factor governing volatile compound alterations (Figure 5E). The color gradient showed that most volatiles increased obviously after storage (blue to red), indicating significant accumulation during aging. In addition, nitrogen treatments did not form distinct subgroups within the same storage period, suggesting that nitrogen levels had a limited effect on the global volatile metabolic pattern.

The volatile substances, key compounds screened by the OPLS-DA model with VIP values greater than 1 and *p* values less than 0.05 were listed in Table 5, including aldehydes and alcohols with obvious odor characteristics, such as Nonanal, Decanal, 1-Octanol, and 1-Nonanol. These substances not only showed a significant upregulated or downregulated trend during storage but also were closely related to the flavor changes in rice, thus being regarded as key differential substances. Their effects on flavor were mainly reflected in fatty acid oxidation and off-flavor formation, with strong sensory impacts. Regarding nitrogen effects, several volatile compounds responded to nitrogen fertilizer application levels. Specifically, alcohols such as 1-octanol and 1-nonanol increased with higher nitrogen levels in certain cultivars; meanwhile, aldehydes including nonanal and decanal accumulated under high nitrogen conditions. This suggested that nitrogen fertilization may promote lipid oxidation, thereby enhancing the formation of volatile aldehydes. Nevertheless, compared with storage duration, the nitrogen effect was considerably weaker.

Combined with the key differential metabolite analysis, the significantly upregulated substances during storage were mainly aldehydes and alcohols, such as (*E*)-2-octenal, nonanal, decanal, and 1-octanol. Most of these are lipid oxidation products characterized by fatty, oily, or aged odors, reflecting intensified fatty acid oxidation during storage. Moreover, increases in compounds such as 1-octen-3-ol were closely associated with off-flavor formation, further explaining the deterioration of rice cooking and eating quality (CEQ) and flavor after long-term storage (Table 5).

In conclusion, storage duration acted as the primary driving factor reshaping volatile profiles, while nitrogen fertilizer application modulated only a part of the volatile metabolites with an overall weak effect. Obvious genotypic differences existed among the tested *indica*-*japonica* hybrid rice cultivars.

## 4. Discussion

Rice quality is a complex trait jointly regulated by genetic and environmental factors, among which cultivar genetic background and grain composition serve as the fundamental determinants, while nitrogen fertilizer application rate, as a key agronomic measure, plays a crucial role in modulating rice quality [58]. Nitrogen is not only an essential nutrient for rice growth and yield formation [59], but also exerts a profound impact on rice quality by modulating the accumulation, distribution and interactions of grain components such as protein, starch, and lipids [60]. Previous studies have consistently demonstrated that nitrogen fertilizer application exerts a dual effect on rice quality, in other words, appropriate nitrogen fertilizer application can improve rice quality, while excessive nitrogen input tends to cause quality deterioration [61,62]. Many previous studies have investigated nitrogen application and storage time on rice quality separately, and zero nitrogen input were used as check [23,24,28,30,31,32,63,64,65,66]. Interestingly, the nitrogen effect was weakened in this study with 4 nitrogen rates of 75, 150, 225 and 300 kg hm^−2^ respectively. In particular, the effects of storage duration, variety, and their interactions among these 3 factors were all token into account. Nitrogen doses only served as the leading factor on HRY, CP and PV (Tables S1–S8). CF, FAV, GC, mostly profiles of RVA, HD, SP, GU and Fb1 were majorly affected postharvest storage. PGWC, AC, ASV, AD, CO and CH were governed by both variety and storage duration. It is worth to mentioning that the interaction effects among nitrogen, variety and storage play a vital role on variation in amylopectin chain-length distribution parameters (Table S8).

Except CO and SP, nitrogen fertilizer application rate exerted significant effects on milling quality, chalkiness, nutrition, CEQ, texture and RVA parameters (Tables S1–S8). With increasing nitrogen fertilizer application rates, MRY increased significantly and reached its maximum under N20 treatment (Table 1). In contrast, the HRY of JFY2 showed a decreasing trend with increasing nitrogen supply. In general, PGWC of three varieties increased with the rising of nitrogen doses, which could be attributed to the excessive filling of the protein matrix promoted by high nitrogen fertilizer application, resulting in the encapsulation and compression of polyhedral starch granules. While local depressions and micropore structures were observed in fresh grains under N20 treatment, indicating that high nitrogen might disrupt the coordinated accumulation of starch and protein, leading to insufficient granule development and leading to the formation of potential structural defects, thereby inducing chalkiness formation (Figure 4). Previous studies have also demonstrated that appropriate nitrogen supply promotes grain filling and dry matter accumulation, thereby enhancing milling and appearance quality, whereas excessive nitrogen application often results in uneven grain filling and increased chalkiness [17,18,19,20].

The rapid decrease in GC induced by the increase in nitrogen fertilizer application level is likely the main reason for the deterioration of CEQ under high nitrogen conditions. GC exhibited significant negative correlations with AC, SB, HD, and CH, while showing positive correlations with PV, BD, and AD; these correlation patterns collectively contributed to the deterioration of rice eating quality (Figure 3). An increase in nitrogen rate markedly elevated the relative content of Fa, indicating that high nitrogen treatment promoted a higher branching density of amylopectin, while amylopectin short chains (DP ≤ 36) could decrease the hardness values [21]. Meanwhile, the relative crystallinity increased with the increasing nitrogen rates, and the starch granules became smaller with an increase in uneven and pitted surfaces (Figure 4). All results indicated that increasing nitrogen altered the structure and properties of starch, eventually resulting in lower GC, thereby affecting texture and starch functional properties and leading to a deterioration in eating quality [67]. In addition, GC also performed significant negative correlations with PGWC and CP, opposite to CF. Usually, rice with low CP and high CF exhibits good eating quality [22,68]. According to our results, when N input exceeded 225 kg hm^−2^, grain protein was highly likely to surpass 8% (Figure 1). In addition, protein contend showed negative correlation with GC, AD and PV (Figure 3). Therefore, 225 kg hm^−2^ could be regarded as the reasonable nitrogen application threshold. After storage, GC further decreased, especially under high nitrogen fertilizer application level, which aggravated the deterioration of eating quality. Therefore, varieties that maintain stable GC or show only a slight decrease in GC under high nitrogen conditions may reduce the risk of quality deterioration. In other words, breeding varieties with high GC under high nitrogen conditions is conducive to the coordinated improvement of yield, appearance quality and eating quality.

From the perspective of compositional regulation, this study demonstrated that crude protein content increased significantly with increasing nitrogen fertilizer application, consistent with previous findings [63,69], Moreover, cultivar-dependent responses to nitrogen were observed, with YY15 and ZJY1578 exhibiting a pronounced increase in protein content under high nitrogen (N20) (Figure 1). Excessive protein accumulation enhances the encapsulation of starch granules, thereby restricting water diffusion into the granules and inhibiting starch swelling and gelatinization, ultimately resulting in increased rice hardness and reduced adhesiveness [70]. High nitrogen application elevated rice protein content and facilitates the adhesion of protein bodies to amyloplast surfaces, mainly in the outer endosperm; meanwhile, protein concentrated around starch to form protein encapsulation further reduced starch retention, thereby suppressing starch gelatinization during cooking [31,71]. Therefore, the deterioration of CEQ under high nitrogen in this study may be attributed not only to increased crude protein content, but also to the altered spatial organization of protein and starch within the endosperm. Scanning electron microscopy (SEM) further revealed that high nitrogen promoted protein deposition on the surface and within the interstitial spaces of starch granules in freshly harvested grains, forming a dense composite structure (Figure 4). Although this structure may improve grain mechanical strength and milling quality to some extent [72], excessive nitrogen disrupts grain filling and induces structural defects [64]. During storage, such high-protein structures are more susceptible to microcrack formation under moisture migration and internal stress, leading to increased chalkiness and reduced processing stability, thereby confirming the poor storability of high-protein rice.

From the perspective of starch structural evolution, changes in starch fine structure constitute a key intrinsic factor underlying quality deterioration. The chain-length distribution of amylopectin indicated that high nitrogen increased the proportion of short chains (F_a_), which, although enhancing branching density, reduced granule structural stability (Table 1). A similar response was observed by Yang X. et al. [65], who found that excessive nitrogen application increased the proportion of short chains in amylopectin, promoted protein particle accumulation in the endosperm, and impaired starch granule development in super hybrid *indica* rice. In addition, increasing nitrogen input decreased amylose content, increased protein content and altered gelatinization characteristics of rice flour, further confirming that nitrogen fertilization can regulate rice quality through starch and protein remodeling [73]. These results help explain the decreased GC and altered RVA parameters observed under high nitrogen treatments in the present study. This observation is consistent with the surface damage of starch granules observed by SEM and further contributes to the deterioration of pasting properties and the reduction in RVA peak viscosity. Meanwhile, during storage, free radicals generated from starch rearrangement and degradation can induce protein oxidation. In addition, starch–lipid complexes are formed via interactions between hydroxyl and carboxyl groups, reducing starch hydrophilicity and increasing the apparent amylose content [66]. In this study, amylose content increased with storage time (Figure 2), although the extent of change may vary depending on cultivar and storage conditions [74]. Consistent with previous studies, rice aging increases amylose content but reduces total starch, lipid, and protein contents, along with the degradation of short-range ordered starch structure and protein secondary structure [37]. Long-term storage also degrades starch molecules, reduces amylopectin long branch chains, double helices, short-range order and crystallinity, and causes obvious erosion of starch granules; prolonged storage likewise lowers starch crystallinity and granule size while elevating the proportion of amylopectin A chains [42,43]. Accordingly, the elevated amylose content and deteriorated RVA and texture properties observed here are closely associated with storage-induced degradation and rearrangement of starch multiscale structures. The combined effects of increased amylose content and elevated fatty acid values contributed to increased hardness and decreased adhesiveness of cooked rice during the later stages of storage [36,75].

Lipid oxidation is a major chemical process driving quality deterioration during storage. With prolonged storage, fatty acid values continuously increased [76,77], indicating ongoing lipid oxidation and degradation. This process not only generates aldehydes and ketones as volatile compounds but also alters the microenvironment of the starch–protein matrix, inducing physical damage such as surface roughening and pitting of starch granules, which is consistent with SEM observations.

Furthermore, a significant interaction between nitrogen fertilizer application and storage duration was observed. The initial structural differences formed under different nitrogen levels are progressively amplified during storage. High nitrogen accelerates lipid oxidation and structural degradation by increasing protein content and altering the grain microenvironment. Even with relatively low initial crude fat content, high-nitrogen treatments exhibited more pronounced quality deterioration [78]. PCA indicated that 12 months of storage represents a critical transition point in the volatile profile, with high nitrogen significantly promoting the accumulation of off-flavor compounds such as nonanal, decanal, (*E*)-2-octenal, and 1-octen-3-ol, ultimately leading to flavor deterioration (Figure 5).

## 5. Conclusions

Nitrogen fertilizer application levels, cultivars and storage duration exert profound and interactive effects on the quality of *indica*-*japonica* hybrid rice. Increasing nitrogen application rate aggravated rice quality deterioration by altering amylopectin fine structure, inducing starch granule surface defects and uneven morphology, and promoting the accumulation of off-flavor volatile alcohols and aldehydes during prolonged storage. These changes jointly reduced gel consistency and altered RVA pasting properties and cooked rice texture, ultimately deteriorating eating and sensory quality. High crude protein content under excessive nitrogen input facilitated protein deposition around starch granules during cooking, which restricted starch gelatinization and further impaired eating quality. GC could therefore serve as an indirect indicator for evaluating rice deterioration and be applied in the breeding of rice varieties with improved storage tolerance. To optimize the comprehensive quality and storage tolerance of *indica*-*japonica* hybrid rice, nitrogen fertilizer application should be maintained at a moderate level (ideally ≤225 kg hm^−2^). The precise fertilizer application rates for different varieties, while balancing yield and storage stability, still need to be further optimized and determined in future.

## Figures and Tables

**Table 1:** Effects of nitrogen fertilizer application on processing and appearance quality of *indica*-*japonica* hybrid rice under different storage durations.

S	N	MRY (%)			HRY (%)			PGWC (%)		
		JFY2	YY15	ZJY1578	JFY2	YY15	ZJY1578	JFY2	YY15	ZJY1578
0M	N5	70.95 ± 0.23 def	75.99 ± 0.31 abc	69.65 ± 0.37 bcd	62.19 ± 0.71 a	59.70 ± 2.03 bcde	58.10 ± 0.34 cd	28.89 ± 1.69 efg	31.24 ± 3.14 c	47.61 ± 3.24 cd
	N10	71.85 ± 0.73 cd	76.16 ± 0.58 abc	69.73 ± 0.19 bc	50.73 ± 3.08 e	56.37 ± 0.20 de	59.14 ± 1.13 bcd	27.86 ± 1.80 fg	39.32 ± 2.82 b	35.98 ± 2.32 e
	N15	72.47 ± 0.54 bc	75.72 ± 0.71 c	70.54 ± 0.72 ab	54.05 ± 1.47 d	55.86 ± 2.15 e	60.51 ± 1.26 bc	30.17 ± 2.38 efg	41.32 ± 3.10 b	38.59 ± 1.74 e
	N20	73.62 ± 0.76 a	77.03 ± 0.35 a	71.43 ± 0.62 a	58.46 ± 1.54 bc	64.55 ± 2.36 a	61.25 ± 0.13 ab	33.33 ± 3.04 d	41.62 ± 1.58 b	44.74 ± 2.00 d
6M	N5	70.67 ± 0.03 efg	75.47 ± 0.80 c	69.05 ± 0.54 cd	60.43 ± 0.51 ab	61.42 ± 0.70 abc	58.03 ± 1.27 cd	27.11 ± 1.38 g	34.73 ± 2.09 c	47.25 ± 1.14 cd
	N10	70.15 ± 0.52 fg	76.20 ± 0.37 abc	68.65 ± 0.49 cd	57.26 ± 2.00 c	57.96 ± 2.16 cde	57.76 ± 0.84 cd	28.12 ± 0.86 fg	24.27 ± 4.14 d	49.36 ± 1.60 c
	N15	71.63 ± 0.74 cde	75.62 ± 0.82 c	67.21 ± 0.83 e	54.11 ± 1.24 d	57.71 ± 0.65 cde	59.38 ± 1.49 bcd	30.54 ± 2.09 def	27.69 ± 1.19 d	47.90 ± 2.99 c
	N20	73.44 ± 0.85 ab	76.98 ± 0.31 ab	69.41 ± 0.77 cd	60.42 ± 0.39 ab	62.84 ± 1.45 ab	63.47 ± 0.54 a	31.59 ± 1.82 de	25.98 ± 1.54 d	57.18 ± 2.00 b
12M	N5	69.99 ± 0.88 fg	75.45 ± 0.03 c	68.48 ± 0.5 d	58.83 ± 0.42 bc	58.82 ± 2.31 bcde	57.33 ± 1.49 de	37.78 ± 2.34 c	39.33 ± 1.20 b	58.44 ± 1.50 ab
	N10	69.81 ± 0.45 g	76.48 ± 0.44 abc	68.66 ± 0.95 cd	50.96 ± 2.41 e	50.66 ± 4.05 f	54.83 ± 2.60 e	52.56 ± 0.84 a	49.23 ± 3.11 a	56.84 ± 0.50 b
	N15	71.22 ± 0.23 de	75.79 ± 1.31 bc	66.61 ± 0.57 e	49.72 ± 1.54 e	51.53 ± 3.98 f	58.91 ± 2.66 bcd	42.05 ± 1.08 b	39.66 ± 1.15 b	60.68 ± 0.88 a
	N20	72.96 ± 0.19 ab	76.62 ± 0.26 abc	69.34 ± 0.38 cd	59.34 ± 1.62 abc	60.64 ± 1.84 abcd	60.01 ± 2.13 bcd	44.67 ± 1.77 b	45.78 ± 2.27 a	56.00 ± 2.41 b

Note: Data are expressed as mean ± standard deviation (*n* = 3); different lowercase letters in the same column indicate significant differences at the 0.05 level (Duncan’s range test). 0M, 6M, and 12M represent storage duration for 0, 6, and 12 months, respectively. MRY, HRY and PGWC represent milled rice yield, head rice yield and percentage of grains with chalkiness, respectively. N5, N10, N15, and N20 in the table represent pure nitrogen fertilizer application levels of 75, 150, 225, and 300 kg hm^−2^.

**Table 2:** Effects of nitrogen fertilizer application level on starch gelatinization characteristics of *indica*-*japonica* hybrid rice under different storage durations.

V	S	N	PV (cP)	TV (cP)	BD (cP)	FV (cP)	SB (cP)	PT (°C)
JFY2	0M	N5	3344 ± 57 b	1254 ± 5 e	2090 ± 53 b	2241 ± 47 g	−1104 ± 10 f	79.45 ± 0.05 g
		N10	3590 ± 11 a	1360 ± 40 d	2230 ± 31 a	2437 ± 26 e	−1153 ± 24 f	79.52 ± 0.03 fg
		N15	3329 ± 31 b	1284 ± 31 e	2045 ± 62 b	2323 ± 66 f	−1006 ± 89 e	79.58 ± 0.08 f
		N20	2833 ± 30 g	1142 ± 47 f	1691 ± 50 c	2151 ± 15 h	−682 ± 28 d	78.75 ± 0.09 h
	6M	N5	3231 ± 22 cd	1758 ± 56 b	1473 ± 35 d	2925 ± 28 cd	−306 ± 50 c	80.35 ± 0.05 e
		N10	3248 ± 42 c	1757 ± 52 b	1491 ± 43 d	2994 ± 9 b	−253 ± 36 c	81.12 ± 0.03 c
		N15	3213 ± 13 cd	1663 ± 62 c	1550 ± 67 d	2930 ± 34 cd	−283 ± 33 c	80.37 ± 0.06 e
		N20	2841 ± 63 g	1678 ± 26 c	1163 ± 72 f	2893 ± 19 d	52 ± 81 b	79.52 ± 0.08 fg
	12M	N5	3121 ± 22 ef	1854 ± 54 a	1267 ± 58 e	3334 ± 56 a	213 ± 40 a	81.55 ± 0.09 a
		N10	3170 ± 25 de	1925 ± 22 a	1245 ± 45 ef	3373 ± 24 a	203 ± 47 a	81.37 ± 0.03 b
		N15	3086 ± 27 f	1874 ± 38 a	1213 ± 54 ef	3326 ± 31 a	240 ± 54 a	81.53 ± 0.03 a
		N20	2730 ± 47 h	1682 ± 28 c	1048 ± 55 g	2962 ± 21 bc	232 ± 36 a	80.82 ± 0.06 d
YY15	0M	N5	3633 ± 36 b	1377 ± 11 f	2256 ± 26 b	2338 ± 20 g	−1295 ± 55 h	78.63 ± 0.03 c
		N10	3774 ± 24 a	1323 ± 26 g	2451 ± 44 a	2261 ± 56 h	−1513 ± 76 i	78.00 ± 0.10 e
		N15	3348 ± 41 c	1267 ± 29 h	2080 ± 20 c	2236 ± 22 h	−1111 ± 63 g	78.28 ± 0.41 d
		N20	3332 ± 51 c	1311 ± 47 g	2021 ± 78 d	2263 ± 16 h	−1069 ± 37 g	77.70 ± 0.13 f
	6M	N5	3346 ± 19 c	1854 ± 16 c	1492 ± 36 f	2946 ± 4 d	−399 ± 16 e	79.25 ± 0.23 b
		N10	3305 ± 22 c	1717 ± 8 e	1589 ± 17 e	2823 ± 8 e	−482 ± 29 f	79.47 ± 0.06 b
		N15	3233 ± 16 d	1719 ± 18 e	1514 ± 17 f	2752 ± 48 f	−482 ± 64 f	79.47 ± 0.10 b
		N20	3180 ± 44 e	1880 ± 14 c	1299 ± 30 g	2951 ± 8 d	−229 ± 36 d	79.48 ± 0.08 b
	12M	N5	3253 ± 18 d	2117 ± 25 a	1136 ± 18 h	3319 ± 13 a	65 ± 30 b	80.58 ± 0.06 a
		N10	3050 ± 24 fg	1863 ± 14 c	1187 ± 28 h	3111 ± 11 c	61 ± 28 b	80.73 ± 0.03 a
		N15	3004 ± 8 g	1812 ± 29 d	1192 ± 27 h	2925 ± 13 d	−78 ± 14 c	80.78 ± 0.03 a
		N20	3065 ± 26 f	2016 ± 7 b	1048 ± 31 i	3205 ± 10 b	140 ± 25 a	80.85 ± 0.05 a
ZJY1578	0M	N5	3552 ± 58 a	1578 ± 54 b	1974 ± 109 ab	2622 ± 88 e	−929 ± 132 g	80.32 ± 0.33 ab
		N10	3321 ± 27 b	1299 ± 13 de	2022 ± 33 a	2356 ± 33 f	−965 ± 43 g	78.75 ± 0.18 d
		N15	3097 ± 12 c	1229 ± 28 e	1868 ± 15 ab	2219 ± 15 g	−878 ± 21 g	78.78 ± 0.32 d
		N20	2770 ± 44 d	1128 ± 28 f	1642 ± 71 c	2112 ± 22 h	−658 ± 63 f	77.98 ± 0.65 e
	6M	N5	3482 ± 35 a	1651 ± 67 b	1830 ± 40 b	2839 ± 25 b	−643 ± 15 f	80.43 ± 0.19 a
		N10	3290 ± 53 b	1476 ± 123 c	1814 ± 174 b	2777 ± 35 c	−512 ± 18 e	79.48 ± 0.08 c
		N15	3072 ± 16 c	1480 ± 47 c	1592 ± 43 c	2693 ± 8 d	−379 ± 10 d	79.95 ± 0.30 bc
		N20	2780 ± 84 d	1370 ± 51 d	1410 ± 135 d	2625 ± 26 e	−155 ± 66 c	79.55 ± 0.05 c
	12M	N5	3007 ± 9 c	1767 ± 11 a	1239 ± 12 e	2959 ± 17 a	−47 ± 22 c	80.82 ± 0.19 a
		N10	2856 ± 64 d	1650 ± 11 b	1206 ± 64 e	2942 ± 43 a	86 ± 82 b	80.73 ± 0.16 a
		N15	2537 ± 47 e	1614 ± 6 b	923 ± 42 f	2870 ± 27 b	333 ± 74 a	80.70 ± 0.05 a
		N20	2509 ± 148 e	1628 ± 6 b	881 ± 151 f	2849 ± 12 b	339 ± 148 a	80.57 ± 0.13 a

Note: PV, TV, BD, FV, SB, and PT represent peak viscosity, trough viscosity, breakdown, final viscosity, setback, and pasting temperature, respectively. N5, N10, N15, and N20 in the table represent pure nitrogen fertilizer application rates of 75, 150, 225, and 300 kg hm^−2^. Different letters within one cultivar represent significant differences at *p* < 0.05, *n* = 3.

**Table 3:** Effects of nitrogen fertilizer application level on texture characteristics of *indica*-*japonica* hybrid rice under different storage durations.

V	S	N	HD (N)	AD (N·s)	CO	SP (mm)	GU (N)	CH (mj)
JFY2	0M	N5	5.49 ± 0.17 g	0.44 ± 0.07 a	0.40 ± 0.01 a	0.84 ± 0.03 ab	2.21 ± 0.12 g	1.86 ± 0.05 d
		N10	5.54 ± 0.10 g	0.43 ± 0.01 a	0.38 ± 0.02 a	0.80 ± 0.03 abcd	2.12 ± 0.08 g	1.69 ± 0.08 d
		N15	5.47 ± 0.10 g	0.32 ± 0.02 bc	0.40 ± 0.02 a	0.85 ± 0.07 a	2.18 ± 0.13 g	1.85 ± 0.04 d
		N20	5.60 ± 0.14 g	0.43 ± 0.01 a	0.37 ± 0.01 ab	0.84 ± 0.03 ab	2.07 ± 0.06 g	1.74 ± 0.09 d
	6M	N5	8.83 ± 0.39 e	0.33 ± 0.01 bc	0.37 ± 0.03 ab	0.77 ± 0.03 cd	3.28 ± 0.30 de	2.54 ± 0.29 b
		N10	9.32 ± 0.14 d	0.36 ± 0.01 b	0.37 ± 0.02 ab	0.76 ± 0.05 d	3.41 ± 0.18 cde	2.61 ± 0.28 b
		N15	8.45 ± 0.14 e	0.31 ± 0.01 c	0.37 ± 0.02 ab	0.82 ± 0.03 abc	3.11 ± 0.16 e	2.55 ± 0.08 b
		N20	6.70 ± 0.36 f	0.29 ± 0.03 cd	0.38 ± 0.02 a	0.79 ± 0.01 bcd	2.57 ± 0.10 f	2.02 ± 0.08 cd
	12M	N5	11.17 ± 0.71 bc	0.25 ± 0.04 d	0.34 ± 0.02 bc	0.69 ± 0.03 f	3.78 ± 0.39 ab	2.62 ± 0.39 b
		N10	11.63 ± 0.47 ab	0.33 ± 0.02 bc	0.34 ± 0.02 bc	0.75 ± 0.02 de	3.93 ± 0.30 a	2.95 ± 0.19 a
		N15	11.74 ± 0.08 a	0.31 ± 0.03 c	0.32 ± 0.00 c	0.70 ± 0.04 ef	3.75 ± 0.07 abc	2.62 ± 0.14 b
		N20	11.10 ± 0.09 c	0.32 ± 0.02 bc	0.32 ± 0.04 c	0.65 ± 0.04 f	3.52 ± 0.37 bcd	2.32 ± 0.35 bc
YY15	0M	N5	5.75 ± 0.32 g	0.45 ± 0.04 a	0.38 ± 0.01 a	0.85 ± 0.03 ab	2.21 ± 0.18 cd	1.89 ± 0.21 bcd
		N10	5.93 ± 0.10 g	0.44 ± 0.03 ab	0.36 ± 0.04 a	0.80 ± 0.05 c	2.13 ± 0.25 cd	1.71 ± 0.22 de
		N15	6.00 ± 0.35 g	0.30 ± 0.03 de	0.37 ± 0.03 a	0.81 ± 0.02 bc	2.22 ± 0.05 cd	1.79 ± 0.07 cde
		N20	6.10 ± 0.58 g	0.38 ± 0.07 bc	0.38 ± 0.02 a	0.88 ± 0.07 a	2.34 ± 0.32 cd	2.06 ± 0.15 b
	6M	N5	6.91 ± 0.13 ef	0.39 ± 0.04 abc	0.30 ± 0.01 b	0.70 ± 0.01 de	2.06 ± 0.11 d	1.45 ± 0.09 f
		N10	6.63 ± 0.20 f	0.36 ± 0.01 cd	0.31 ± 0.02 b	0.71 ± 0.04 d	2.05 ± 0.16 d	1.46 ± 0.08 f
		N15	7.25 ± 0.30 e	0.28 ± 0.03 e	0.32 ± 0.02 b	0.71 ± 0.02 de	2.27 ± 0.17 cd	1.60 ± 0.10 ef
		N20	8.98 ± 0.31 d	0.31 ± 0.08 de	0.31 ± 0.03 b	0.73 ± 0.02 d	2.79 ± 0.34 b	2.04 ± 0.25 b
	12M	N5	9.96 ± 0.33 c	0.29 ± 0.02 de	0.24 ± 0.02 c	0.62 ± 0.04 f	2.35 ± 0.22 cd	1.46 ± 0.06 f
		N10	9.75 ± 0.20 c	0.33 ± 0.02 cde	0.25 ± 0.03 c	0.70 ± 0.01 de	2.46 ± 0.23 c	1.72 ± 0.13 de
		N15	12.92 ± 0.32 b	0.27 ± 0.02 e	0.24 ± 0.00 c	0.66 ± 0.04 ef	3.06 ± 0.11 b	2.02 ± 0.15 bc
		N20	14.00 ± 0.39 a	0.31 ± 0.01 de	0.25 ± 0.01 c	0.68 ± 0.01 de	3.50 ± 0.18 a	2.38 ± 0.13 a
ZJY1578	0M	N5	5.47 ± 0.32 hi	0.38 ± 0.01 a	0.37 ± 0.04 a	0.86 ± 0.02 a	2.04 ± 0.13 fg	1.76 ± 0.16 c
		N10	5.20 ± 0.41 i	0.38 ± 0.04 a	0.36 ± 0.04 ab	0.80 ± 0.02 b	1.88 ± 0.37 g	1.52 ± 0.33 c
		N15	5.29 ± 0.21 i	0.35 ± 0.07 a	0.36 ± 0.02 ab	0.79 ± 0.03 bcd	1.92 ± 0.11 g	1.52 ± 0.10 c
		N20	6.00 ± 0.15 h	0.31 ± 0.02 b	0.37 ± 0.03 ab	0.77 ± 0.03 bcdef	2.20 ± 0.20 efg	1.70 ± 0.22 c
	6M	N5	6.89 ± 0.51 g	0.28 ± 0.01 b	0.34 ± 0.03 ab	0.75 ± 0.02 def	2.35 ± 0.20 def	1.77 ± 0.17 c
		N10	7.59 ± 0.24 ef	0.27 ± 0.02 b	0.32 ± 0.04 bc	0.74 ± 0.02 ef	2.46 ± 0.32 de	1.81 ± 0.21 c
		N15	7.10 ± 0.45 fg	0.22 ± 0.04 c	0.38 ± 0.02 a	0.80 ± 0.02 bc	2.72 ± 0.18 cd	2.17 ± 0.15 b
		N20	7.67 ± 0.24 e	0.19 ± 0.03 c	0.39 ± 0.02 a	0.78 ± 0.04 bcde	2.98 ± 0.23 bc	2.33 ± 0.24 b
	12M	N5	9.91 ± 0.05 d	0.19 ± 0.01 c	0.22 ± 0.01 e	0.75 ± 0.03 def	2.22 ± 0.08 efg	1.67 ± 0.11 c
		N10	11.33 ± 0.28 c	0.20 ± 0.03 c	0.29 ± 0.03 cd	0.75 ± 0.03 def	3.27 ± 0.42 ab	2.45 ± 0.40 ab
		N15	13.22 ± 0.09 a	0.20 ± 0.02 c	0.27 ± 0.01 d	0.76 ± 0.04 cdef	3.57 ± 0.09 a	2.70 ± 0.22 a
		N20	12.33 ± 0.68 b	0.19 ± 0.02 c	0.26 ± 0.03 de	0.73 ± 0.01 f	3.15 ± 0.20 b	2.29 ± 0.16 b

Note: HD, AD, CO, SP, GU and CH represent hardness, adhesiveness, cohesiveness, springiness, gumminess and chewiness, respectively. N5, N10, N15, and N20 in the table represent pure nitrogen fertilizer application rates of 75, 150, 225, and 300 kg hm^−2^. Different letters within one cultivar represent significant differences at *p* < 0.05, *n* = 3.

**Table 4:** Amylopectin chain-length distribution of *indica*-*japonica* hybrid rice after storage under different nitrogen fertilizer application levels.

V	S	N	Fa (%)	Fb_1_ (%)	Fb_2_ (%)	Fb_3_ (%)	ACL (DP)
JFY2	0M	N5	26.81 ± 0.16 b	49.64 ± 0.05 e	11.46 ± 0.22 a	12.09 ± 0.21 c	20.28 ± 0.09 b
		N10	25.58 ± 0.13 e	50.45 ± 0.19 bc	11.48 ± 0.13 a	12.49 ± 0.13 a	20.60 ± 0.14 a
		N15	25.58 ± 0.21 e	50.56 ± 0.13 b	11.46 ± 0.11 a	12.39 ± 0.12 a	20.56 ± 0.14 a
		N20	25.68 ± 0.03 e	50.35 ± 0.11 c	11.48 ± 0.13 a	12.49 ± 0.13 a	20.61 ± 0.25 a
	12M	N5	25.99 ± 0.06 d	50.33 ± 0.23 cd	11.35 ± 0.22 ab	12.33 ± 0.11 ab	20.56 ± 0.15 a
		N10	26.17 ± 0.15 c	50.19 ± 0.06 d	11.36 ± 0.13 ab	12.29 ± 0.11 abc	20.58 ± 0.19 a
		N15	26.21 ± 0.09 c	50.27 ± 0.06 d	11.31 ± 0.23 b	12.21 ± 0.14 abc	20.52 ± 0.16 a
		N20	28.14 ± 0.05 a	50.77 ± 0.07 a	10.58 ± 0.13 c	10.52 ± 0.06 d	19.74 ± 0.18 c
YY15	0M	N5	25.01 ± 0.15 cd	50.58 ± 0.05 b	11.57 ± 0.21 bc	12.84 ± 0.08 a	20.79 ± 0.22 a
		N10	24.94 ± 0.04 d	50.49 ± 0.11 b	11.70 ± 0.11 ab	12.87 ± 0.14 a	20.82 ± 0.12 a
		N15	25.19 ± 0.09 c	50.56 ± 0.05 b	11.61 ± 0.14 bc	12.65 ± 0.04 b	20.78 ± 0.24 a
		N20	24.68 ± 0.06 e	50.66 ± 0.19 b	11.67 ± 0.12 b	12.99 ± 0.17 a	20.92 ± 0.23 a
	12M	N5	25.84 ± 0.12 a	50.44 ± 0.13 b	11.33 ± 0.22 cd	12.39 ± 0.16 c	20.58 ± 0.23 ab
		N10	25.82 ± 0.05 a	50.54 ± 0.12 b	11.40 ± 0. 23 d	12.24 ± 0.13 c	20.56 ± 0.14 b
		N15	25.68 ± 0.15 ab	50.60 ± 0.06 b	11.31 ± 0.12 d	12.41 ± 0.15 c	20.57 ± 0.18 b
		N20	22.13 ± 0.23 f	53.05 ± 0.21 a	11.93 ± 0.21 a	12.89 ± 0.24 a	20.53 ± 0.09 b
ZJY1578	0M	N5	25.51 ± 0.11 e	50.47 ± 0.04 b	11.36 ± 0.12 ab	12.66 ± 0.12 a	20.67 ± 0.12 a
		N10	25.93 ± 0.13 d	50.30 ± 0.03 bc	11.35 ± 0.03 ab	12.42 ± 0.12 bc	20.57 ± 0.13 a
		N15	26.94 ± 0.02 b	49.54 ± 0.20 d	11.18 ± 0.24 b	12.34 ± 0.03 bc	20.64 ± 0.13 a
		N20	28.13 ± 0.16 a	49.18 ± 0.12 e	11.10 ± 0.12 b	11.59 ± 0.15 e	20.14 ± 0.15 b
	12M	N5	25.97 ± 0.11 d	50.47 ± 0.13 b	11.21 ± 0.21 ab	12.36 ± 0.04 bc	20.56 ± 0.25 a
		N10	26.05 ± 0.05 d	50.49 ± 0.22 b	11.18 ± 0.12 b	12.29 ± 0.17 cd	20.51 ± 0.15 a
		N15	26.62 ± 0.02 c	51.38 ± 0.12 a	11.42 ± 0.14 a	12.59 ± 0.12 a	20.54 ± 0.17 a
		N20	26.42 ± 0.03 c	50.23 ± 0.15 c	11.19 ± 0.11 b	12.17 ± 0.12 d	20.42 ± 0.20 a

Note: Fa, Fb_1_, Fb_2_ and Fb_3_ represent the short A chains, B1 chains, B2 chains and B3 long chains of amylopectin, respectively; ACL refers to the average chain length of amylopectin. N5, N10, N15, and N20 in the table represent pure nitrogen fertilizer application rates of 75, 150, 225, and 300 kg hm^−2^. Different letters within one cultivar represent significant differences at *p* < 0.05.

**Table 5:** Key differential volatile metabolites and their changes in *indica*-*japonica* hybrid rice during storage under different nitrogen fertilizer application levels.

Cultivar	Compound	Category	Odor Description	Storage Trend	Nitrogen Response
JFY2	1-Octanol	Alcohols	Fatty aroma	↑ (aged odor intensified)	↑ with increasing nitrogen
JFY2	2-Octenal (*E*)	Aldehydes	Oily flavor	↑ (oxidation enhanced)	No obvious regularity
JFY2	1-Nonanol	Alcohols	Fatty aroma	↑ (flavor deterioration)	↑ with increasing nitrogen
JFY2	1-Octen-3-ol	Alcohols	Mushroom flavor	↑ (core off-odor)	More significant under high nitrogen
JFY2	Nonanal	Aldehydes	Fatty/grassy aroma	↑ (lipid oxidation)	↑ with increasing nitrogen
JFY2	2-butyl-1-octanol	Alcohols	Fresh aroma	↓ (fresh aroma loss)	↓ with increasing nitrogen
YY15	Decanal	Aldehydes	Citrus aroma	↑ (oxidation product)	More significant under high nitrogen
YY15	Undecanal	Aldehydes	Fatty aroma	↑ (oxidation enhanced)	No obvious regularity
YY15	Benzaldehyde	Aromatic aldehydes	Almond aroma	↑ (aged flavor formation)	↑ with increasing nitrogen
YY15	1-Octanol	Alcohols	Fatty aroma	↑ (aged odor intensified)	More significant under high nitrogen
YY15	1-Nonanol	Alcohols	Fatty aroma	↑ (quality decline)	↑ with increasing nitrogen
YY15	2-Octenal (*E*)	Aldehydes	Oily flavor	↑ (lipid oxidation)	No obvious regularity
ZJY1578	2-Hexyl-1-octanol	Alcohols	Green aroma	↑ (slight oxidation)	No obvious regularity
ZJY1578	2-Butyl-1-octanol	Alcohols	Fresh aroma	↓ (fresh aroma loss)	↓ with increasing nitrogen

Note: ↑ and ↓ represent the increase and decrease in compound content with storage duration or nitrogen application rate.

## Data Availability

The original contributions presented in this study are included in the article/Supplementary Materials. Further inquiries can be directed to the corresponding author.

## Supplementary Materials

The following supporting information can be downloaded at: https://www.mdpi.com/article/10.3390/foods15101793/s1, Table S1: Analysis of variance for processing and appearance quality traits. Table S2: Analysis of variance for physicochemical quality traits. Table S3: Analysis of variance for cooking quality traits. Table S4: Analysis of variance for pasting properties I. Table S5: Analysis of variance for pasting properties II. Table S6: Analysis of variance for texture properties I. Table S7: Analysis of variance for texture properties II. Table S8: Analysis of variance for amylopectin chain-length distribution parameters.

## Abbreviations

The following abbreviations are used in this manuscript:

MRY	Milled rice yield
HRY	Head rice yield
PGWC	Percentage of grain with chalkiness
CP	Crude protein
CF	Crude fat
FAV	Fatty acid value
AC	Amylose content
GC	Gel consistency
ASV	Alkali spreading value
PV	Peak viscosity
TV	Trough viscosity
BD	Breakdown
FV	Final viscosity
SB	Setback
GT	Gelatinization temperature
HD	Hardness
AD	Adhesiveness
CO	Cohesiveness
SP	Springiness
GU	Gumminess
CH	Chewiness
Fa	Fa fraction (DP 6–12)
Fb_1_	Fb1 fraction (DP 13–24)
Fb_2_	Fb2 fraction (DP 25–36)
Fb_3_	Fb3 fraction (DP ≥ 37)
ACL	Average chain length
