# Supplementary material for: Evolution of Rice Storage Quality and Underlying Microstructural Mechanisms Under Varying Nitrogen Fertilization Application Levels
Source: Foods. 2026 May 19;15(10):1793. doi: 10.3390/foods15101793 (PMC13205652; doi:10.3390/foods15101793)
Supplement: Supplementary file 1 [file foods-15-01793-s001.zip › foods-4292237-supplementary.pdf]

foods

MDPI

Appendix A

Table S1. Analysis of variance for processing and appearance quality traits

Source	DF	MRY				HRY				PGWC			
		SS	MS	F	*P*	SS	MS	F	*P*	SS	MS	F	*P*
Replication	2	1.026	0.513	—	—	5.463	2.731	—	—	7.994	3.997	—	—
N	3	50.316	16.772	28.72**	0.000589	700.559	233.520	113.69**	0.000011	149.954	49.985	26.25**	0.000755
Error A	6	3.504	0.584	—	—	12.322	2.054	—	—	11.427	1.904	—	—
V	2	923.077	461.539	1073.35**	8.97×10^{-18}	134.698	67.349	20.10**	0.000043	5078.369	2539.185	662.14**	4.12×10^{-16}
N × V	6	22.191	3.699	8.60**	0.000277	258.764	43.127	12.87**	0.000024	193.542	32.257	8.41**	0.000314
Error B	16	6.879	0.430	—	—	53.597	3.350	—	—	61.374	3.836	—	—
S	2	25.909	12.954	41.79**	3.08×10^{-11}	207.793	103.897	28.65**	6.48×10^{-9}	3601.979	1800.990	651.12**	1.66×10^{-35}
N × S	6	2.977	0.496	1.60	0.168	45.806	7.634	2.11	0.069	366.993	61.165	22.11**	2.76×10^{-12}
V × S	4	13.120	3.280	10.58**	0.000003	30.626	7.657	2.11	0.094	1148.022	287.005	103.76**	5.34×10^{-23}
N × V × S	12	10.358	0.863	2.78**	0.006	94.009	7.834	2.16*	0.030	828.334	69.028	24.96**	1.36×10^{-16}
Error C	48	14.864	0.310	—	—	174.046	3.626	—	—	132.769	2.766	—	—

Note: N, V, and S represent nitrogen application rate, rice variety, and storage duration, respectively. MRY, HRY, and PGWC represent milled rice yield, head rice yield, and percentage of grains with chalkiness, respectively. SS and MS represent sum of squares and mean square, respectively. * and ** indicate significance at $P< 0.05$ and $P < 0.01$, respectively.

foods

MDPI

Table S2. Analysis of variance for physicochemical quality traits

Source	DF	CP				CF				FAV			
		SS	MS	F	*P*	SS	MS	F	*P*	SS	MS	F	*P*
Replication	2	0.005	0.002	—	—	0.056	0.028	—	—	0.12	0.06	—	—
N	3	25.179	8.393	839.30**	2.94×10^{-8}	0.376	0.125	17.24**	0.002	69.134	23.045	33.35**	3.88×10^{-4}
Error A	6	0.061	0.010	—	—	0.044	0.007	—	—	4.144	0.691	—	—
V	2	18.719	9.359	719.92**	2.13×10^{-16}	0.061	0.030	4.96*	0.021	1767.202	883.601	654.03**	4.61×10^{-16}
N × V	6	5.081	0.847	65.15**	2.40×10^{-10}	0.092	0.015	2.52	0.065	31.505	5.251	3.89*	0.014000
Error B	16	0.209	0.013	—	—	0.098	0.006	—	—	21.621	1.351	—	—
S	2	1.332	0.666	39.18**	8.15×10^{-11}	0.846	0.423	56.14**	2.71×10^{-13}	24733.72	12366.86	6186.52**	1.23×10^{-58}
N × S	6	0.125	0.021	1.24	0.303	0.003	0.0004	0.06	0.999	199.067	33.178	16.60**	3.01×10^{-10}
V × S	4	0.140	0.035	2.06	0.100	0.083	0.021	2.75*	0.039	1812.571	453.143	226.68**	1.62×10^{-30}
N × V × S	12	0.187	0.016	0.92	0.534	0.015	0.001	0.17	0.999	131.817	10.985	5.50**	8.66×10^{-6}
Error C	48	0.798	0.017	—	—	0.362	0.008	—	—	95.961	1.999	—	—

Note: N, V, and S represent nitrogen application rate, rice variety, and storage duration, respectively. CP, CF, and FAV represent crude protein content, crude fat content, and fatty acid value, respectively. SS and MS represent sum of squares and mean square, respectively. * and ** indicate significance at $P < 0.05$ and $P < 0.01$, respectively.

foods

MDPI

Table S3. Analysis of variance for cooking quality traits

Source	DF	AC				GC				ASV			
		SS	MS	F	*P*	SS	MS	F	*P*	SS	MS	F	*P*
Replication	2	0.077	0.039	—	—	48.934	24.467	—	—	0.168	0.084	—	—
N	3	23.125	7.708	42.12**	2.00×10^{-4}	1916.391	638.797	43.40**	1.84×10^{-4}	1.266	0.422	1.90	0.231
Error A	6	1.097	0.183	—	—	88.424	14.737	—	—	1.332	0.222	—	—
V	2	220.722	110.361	276.59**	3.90×10^{-13}	964.856	482.428	20.06**	0.000044	5.779	2.89	16.61**	1.25×10^{-4}
N × V	6	39.683	6.614	16.58**	4.70×10^{-6}	267.547	44.591	1.85	0.153	2.787	0.464	2.67	0.055
Error B	16	6.379	0.399	—	—	384.817	24.051	—	—	2.787	0.174	—	—
S	2	233.401	116.701	328.74**	1.01×10^{-28}	5900.859	2950.430	188.84**	1.79×10^{-23}	7.677	3.839	15.80**	0.000006
N × S	6	25.387	4.231	11.92**	3.81×10^{-8}	57.755	9.626	0.62	0.713	0.386	0.064	0.26	0.952
V × S	4	29.762	7.440	20.96**	4.78×10^{-10}	126.424	31.606	2.02	0.107	0.348	0.087	0.36	0.837
N × V × S	12	18.847	1.571	4.42**	9.99×10^{-5}	244.804	20.400	1.31	0.244	1.309	0.109	0.45	0.934
Error C	48	17.059	0.355	—	—	749.951	15.624	—	—	11.681	0.243	—	—

Note: N, V, and S represent nitrogen application rate, rice variety, and storage duration, respectively. AC, GC, and ASV represent amylose content, gel consistency, and alkali spreading value, respectively. SS and MS represent sum of squares and mean square, respectively. * and ** indicate significance at $P < 0.05$ and $P < 0.01$, respectively.

foods

MDPI

Table S4. Analysis of variance for pasting properties I

Source	DF	PV				TV				BD			
		SS	MS	F	*P*	SS	MS	F	*P*	SS	MS	F	*P*
Replication	2	12550.57	6275.287	—	—	1657.574	828.787	—	—	11966.06	5983.028	—	—
N	3	3238607	1079536	1076.79**	1.39×10^{-8}	390782.5	130260.8	73.78**	3.98×10^{-5}	1773024	591007.900	148.19**	5.14×10^{-6}
Error A	6	6015.278	1002.546	—	—	10593.09	1765.515	—	—	23928.54	3988.090	—	—
V	2	1324055	662027.70	339.48**	7.89×10^{-14}	716536.7	358268.3	216.24**	2.62×10^{-12}	111081.50	55540.750	12.83**	0.000473
N × V	6	877873.4	146312.20	75.03**	8.15×10^{-11}	378349.5	63058.26	38.06**	1.35×10^{-8}	200062.20	33343.700	7.70**	0.000513
Error B	16	31201.93	1950.12	—	—	26509.56	1656.847	—	—	69266.30	4329.144	—	—
S	2	2621558	1310779	662.85**	1.10×10^{-35}	5177795	2588897	1672.91**	4.10×10^{-45}	14634378.00	7317189.000	1841.11**	4.25×10^{-46}
N × S	6	283432.9	47238.82	23.89**	7.24×10^{-13}	44285.93	7380.988	4.77**	0.000693	155323.80	25887.290	6.51**	4.47×10^{-5}
V × S	4	437809.7	109452.4	55.35**	2.17×10^{-17}	381148.6	95287.16	61.57**	2.65×10^{-18}	1022271.00	255567.900	64.30**	1.11×10^{-18}
N × V × S	12	151421.3	12618.44	6.38**	1.36×10^{-6}	147927.6	12327.3	7.97**	6.53×10^{-8}	150234.00	12519.5	3.15**	0.002
Error C	48	94918.89	1977.477	—	—	74281.78	1547.537	—	—	190767.80	3974.329	—	—

Table S5. Analysis of variance for pasting properties II

Source	DF	FV				SB				GT			
		SS	MS	F	*P*	SS	MS	F	*P*	SS	MS	F	*P*
Replication	2	208.296	104.148	—	—	9671.796	4835.898	—	—	0.057	0.029	—	—
N	3	491085.963	163695.321	182.63**	2.77×10^{-6}	1345041.657	448347.219	201.11**	2.08×10^{-6}	6.689	2.230	63.71**	6.10×10^{-5}
Error A	6	5377.926	896.321	—	—	13375.981	2229.330	—	—	0.213	0.035	—	—
V	2	523007.463	261503.731	131.47**	1.17×10^{-10}	893073.019	446536.509	102.08**	7.78×10^{-10}	14.311	7.155	166.40**	1.96×10^{-11}
N × V	6	511822.315	85303.719	42.89**	5.58×10^{-9}	186246.093	31041.015	7.10**	0.0008	5.029	0.838	19.49**	1.58×10^{-6}
Error B	16	31824.667	1989.042	—	—	69989.333	4374.333	—	—	0.688	0.043	—	—
S	2	12086983.69	6043491.843	7869.46**	3.90×10^{-61}	25275907.46	12637953.73	4447.39**	3.27×10^{-55}	79.864	39.932	1331.07**	9.08×10^{-43}
N × S	6	67688.315	11281.386	14.69**	1.94×10^{-9}	255199.426	42533.238	14.97**	1.46×10^{-9}	3.179	0.53	17.67**	1.13×10^{-10}
V × S	4	488884.148	122221.037	159.15**	4.65×10^{-27}	614779.315	153694.829	54.09**	3.41×10^{-17}	2.244	0.561	18.70**	2.53×10^{-9}
N × V × S	12	308130.074	25677.506	33.44**	3.39×10^{-19}	334254.241	27854.520	9.80**	2.87×10^{-9}	3.446	0.287	9.57**	4.20×10^{-9}
Error C	48	36862.444	767.968	—	—	136399.556	2841.657	—	—	1.429	0.030	—	—

Note: N, V, and S represent nitrogen application rate, rice variety, and storage duration, respectively. PV, TV, BD, FV, SB, and GT represent peak viscosity, trough viscosity, breakdown viscosity, final viscosity, setback viscosity, and gelatinization temperature, respectively. SS and MS represent sum of squares and mean square, respectively. * and ** indicate significance at $P < 0.05$ and $P < 0.01$, respectively.

foods

MDPI

Table S6. Analysis of variance for texture properties I

Source	DF	HD				AD				CO			
		SS	MS	F	*P*	SS	MS	F	*P*	SS	MS	F	*P*
Replication	2	0.041	0.021	—	—	0.001	0.001	—	—	0.001	0.0003	—	—
N	3	14.494	4.831	181.54**	2.82×10^{-6}	0.063	0.021	34.71**	0.000346	0.001	0.0003	0.50	0.695
Error A	6	0.16	0.027	—	—	0.004	0.001	—	—	0.004	0.001	—	—
V	2	1.236	0.618	5.12*	0.019	0.149	0.075	97.68**	1.08×10^{-9}	0.057	0.028	69.11**	1.34×10^{-8}
N × V	6	30.141	5.024	41.60**	6.99×10^{-9}	0.029	0.005	6.30**	0.001	0.004	0.001	1.80	0.162
Error B	16	1.932	0.121	—	—	0.012	0.001	—	—	0.007	0.0004	—	—
S	2	654.513	327.256	3492.45**	1.04×10^{-52}	0.27	0.135	175.28**	8.67×10^{-23}	0.185	0.093	191.44**	1.33×10^{-23}
N × S	6	21.703	3.617	38.60**	9.72×10^{-17}	0.042	0.007	9.10**	1.20×10^{-6}	0.009	0.002	3.20*	0.01
V × S	4	8.188	2.047	21.84**	2.57×10^{-10}	0.018	0.005	6.00**	0.000536	0.026	0.006	13.29**	2.32×10^{-7}
N × V × S	12	17.864	1.489	15.89**	7.82×10^{-13}	0.016	0.001	1.72	0.093	0.008	0.001	1.43	0.187
Error C	48	4.498	0.094	—	—	0.037	0.001	—	—	0.023	0.0004	—	—

foods

MDPI

Table S7. Analysis of variance for texture properties II

Source	DF	SP				GU				CH			
		SS	MS	F	*P*	SS	MS	F	*P*	SS	MS	F	*P*
Replication	2	0.006	0.003	—	—	0.036	0.018	—	—	0.057	0.028	—	—
N	3	0.001	0.0002	0.59	0.645	1.406	0.469	9.66*	0.01	0.788	0.263	5.91*	0.032
Error A	6	0.003	0.0004	—	—	0.291	0.049	—	—	0.266	0.044	—	—
V	2	0.028	0.014	10.30**	0.001	5.941	2.971	69.02**	1.35×10^{-8}	4.297	2.149	78.16**	5.53×10^{-9}
N × V	6	0.018	0.003	2.21	0.096	4.193	0.699	16.24**	5.40×10^{-6}	2.737	0.456	16.60**	4.67×10^{-6}
Error B	16	0.022	0.001	—	—	0.689	0.043	—	—	0.44	0.027	—	—
S	2	0.272	0.136	168.80**	1.92×10^{-22}	21.168	10.584	241.86**	8.58×10^{-26}	4.678	2.339	67.87**	1.02×10^{-14}
N × S	6	0.034	0.006	6.93**	2.40×10^{-5}	1.441	0.24	5.49**	0.000217	1.29	0.215	6.24**	6.76×10^{-5}
V × S	4	0.054	0.014	16.80**	1.13×10^{-8}	3.628	0.907	20.72**	5.67×10^{-10}	3.059	0.765	22.19**	2.03×10^{-10}
N × V × S	12	0.019	0.002	1.92	0.055	2.245	0.187	4.27**	0.000141	1.369	0.114	3.31**	0.001522
Error C	48	0.039	0.001	—	—	2.101	0.044	—	—	1.654	0.034	—	—

Note: N, V, and S represent nitrogen application rate, rice variety, and storage duration, respectively. HD, AD, CO, SP, GU, and CH represent hardness, adhesiveness, cohesiveness, springiness, gumminess, and chewiness, respectively. SS and MS represent sum of squares and mean square, respectively. * and ** indicate significance at $P < 0.05$ and $P < 0.01$, respectively.

foods

MDPI

Table S8. Analysis of variance for amylopectin chain-length distribution parameters I

Source	DF	Fa				Fb_1				Fb_2				Fb_3				ACL			
		SS	MS	F	*P*	SS	MS	F	*P*	SS	MS	F	*P*	SS	MS	F	*P*	SS	MS	F	*P*
Replication	2	0.0004	0.0002	—	—	0.006	0.003	—	—	0.002	0.001	—	—	0.002	0.001	—	—	0.028	0.014	—	—
N	3	0.346	0.115	510.38**	1.30×10^{-7}	1.45	0.483	214.30**	1.72×10^{-6}	0.073	0.024	78.28**	3.35×10^{-5}	1.474	0.491	168.52**	3.51×10^{-6}	0.55	0.183	103.21**	1.49×10^{-5}
Error A	6	0.001	0.0002	—	—	0.014	0.002	—	—	0.002	0.0003	—	—	0.017	0.003	—	—	0.011	0.002	—	—
V	2	28	14	8888.96**	4.27×10^{-25}	5.357	2.679	1709.43**	2.22×10^{-19}	1.339	0.669	552.82**	1.71×10^{-15}	3.866	1.933	1519.50**	5.66×10^{-19}	0.878	0.439	310.12**	1.60×10^{-13}
N × V	6	31.556	5.259	3339.25**	7.39×10^{-24}	9.975	1.663	1060.94**	6.99×10^{-20}	1.119	0.186	153.99**	3.08×10^{-13}	3.611	0.602	473.12**	4.34×10^{-17}	0.488	0.081	57.44**	6.24×10^{-10}
Error B	16	0.025	0.002	—	—	0.025	0.002	—	—	0.019	0.001	—	—	0.02	0.001	—	—	0.023	0.001	—	—
S	1	0.11	0.11	52.60**	1.71×10^{-7}	4.43	4.43	3068.06**	7.73×10^{-27}	0.436	0.436	322.87**	2.01×10^{-15}	1.235	1.235	761.92**	1.08×10^{-19}	0.371	0.371	141.64**	1.48×10^{-11}
N × S	3	3.58	1.193	572.39**	1.88×10^{-22}	4.418	1.473	1019.80**	1.99×10^{-25}	0.044	0.015	10.96**	0.0001	0.504	0.168	103.65**	7.13×10^{-14}	0.237	0.079	30.09**	2.66×10^{-8}
V × S	2	7.474	3.737	1792.38**	7.49×10^{-27}	1.268	0.634	439.10**	1.26×10^{-19}	0.32	0.16	118.62**	3.61×10^{-13}	1.239	0.619	382.09**	6.35×10^{-19}	0.236	0.118	44.95**	7.66×10^{-9}
N × V × S	6	25.194	4.199	2013.93**	3.34×10^{-31}	6.465	1.078	746.25**	4.75×10^{-26}	1.151	0.192	142.21**	1.52×10^{-17}	4.813	0.802	494.89**	6.33×10^{-24}	1.069	0.178	68.01**	7.07×10^{-14}
Error C	24	0.05	0.002	—	—	0.035	0.001	—	—	0.032	0.001	—	—	0.039	0.002	—	—	0.063	0.003	—	—

Note: N, V, and S represent nitrogen application rate, rice variety, and storage duration, respectively. Fa, Fb_1, Fb_2, Fb_3, and ACL represent amylopectin chain-length distribution fractions and average chain length, respectively. SS and MS represent sum of squares and mean square, respectively. * and ** indicate significance at $P < 0.05$ and $P < 0.01$, respectively. Only samples stored for 0 and 12 months were included in the analysis of amylopectin chain-length distribution parameters.

 https://doi.org/10.3390/foods15101793
